# Supplementary material for: Growth Performance and Biochemical Profiles of Fairy Shrimp (Streptocephalus sirindhornae) Fed Natural Diets at Low and High Stocking Densities
Source: Biology (Basel). 2026 Jan 7;15(2):117. doi: 10.3390/biology15020117 (PMC12837640; doi:10.3390/biology15020117)
Supplement: Supplementary file 1 [file biology-15-00117-s001.zip › biology-3979849-supplementary.pdf]

Supplementary materials

Table S1. Occurrence and relative frequency (*F*; %) of plankton identified in the gut contents of *Streptocephalus sirindhornae* cultured at two different stocking densities.

Categories	Scientific Name	Low density	*F*	High density	*F*
Cyanophyta	*Anabaena* sp.	–	–	+	8
	Anabaenopsis sp.	+	14	+	4
	Chroococcus sp.	+	8	–	–
	Merismopedia sp.	+	8	+	2
	Microcystis sp.	+	36	+	58
	Oscillatoria sp.	+	2	+	4
	Spirulina sp.	+	2	+	2
Chlorophyta	*Actinastrum* sp.	+	12	+	4
	Ankistrodesmus sp.	+	34	+	24
	Chlorella sp.	+	100	+	100
	Closterium sp.	+	40	+	34
	Cosmarium sp.	+	26	+	24
	Crucigenia sp.	+	4	+	2
	Crucigneiella sp.	+	18	+	14
	Dictyosphaerium sp.	–	–	+	10
	Eudorina sp.	+	4	–	–
	Halosphaera sp.	+	16	+	8
	Hyalotheca sp.	–	–	+	2
	Monoraphidium sp.	+	92	+	92
	Oocystis sp.	+	28	+	36
	Pediastrum sp.1	+	6	+	6
	Pediastrum sp.2	+	10	+	12
	Pediastrum sp.3	+	22	+	12
	Scenedesmus sp.	+	66	+	46
	Selenastrum sp.	+	6	+	4
	Spirogyra sp.	+	28	+	46

	Staurastrum sp.1	+	8	+	4
	Tetraedron sp.1	+	10	+	18
	Tetraedron sp.2	+	14	+	12
	Volvox sp.	+	2	–	–
Euglenophyta	*Euglena* sp.	+	46	+	50
	Phacus sp.1	+	32	+	30
	Phacus sp.2	+	18	+	24
	Phacus sp.3	+	4	+	2
	Phacus sp.4	+	20	+	22
	Stombomonas sp.	+	12	+	18
	Trachelomonas sp.	+	38	+	42
Chrysophyta	*Centritractus* sp.	+	6	+	14
Bacillariophyta	*Bacillaria* sp.	+	36	+	38
	Brachysira sp.	+	22	+	20
	Cyclotella sp.	+	4	+	8
	Diploneis sp.	+	2	–	–
	Frustulia sp.	+	48	+	68
	Gomphonema sp.	+	30	+	28
	Gyrosigma sp.	+	6	+	2
	Navicula sp.	+	64	+	78
	Synedra sp.	+	34	+	18
Pyrrhophyta	*Peridinium* sp.	+	4	+	12
Rotifera	*Brachionus angularis*	+	4	–	–
	Brachionus bidentatus	+	2	+	2
	Brachionus diversicornis	+	4	–	–
	Brachionus falcatus	–	–	+	2
	Brachionus murphyi	+	2	+	4
Copepoda	*Cyclopoid copepod*	–	–	+	8
	**Total number of taxa**	**49**		**48**	

Table S2. Occurrence and relative frequency (*F*; %) of phytoplankton in the culture pond.

Categories	Scientific Name	Day 0	Day 3	Day 6	Day 9	Day 12	Day 15	*F*
Cyanophyta	*Anabaena* sp.	+	+	–	–	+	+	27.78
	Anabaenopsis sp.	+	+	+	+	+	+	61.11
	Chroococcus sp.	−	+	−	−	+	–	11.11
	Merismopedia sp.	−	+	+	−	−	+	16.67
	Microcystis sp.	−	–	+	+	+	+	55.56
	Oscillatoria sp.	−	+	+	–	+	+	50.00
	Spirulina sp.	−	+	+	−	−	+	16.67
Chlorophyta	*Actinastrum* sp.	−	+	+	−	−	+	16.67
	Actinotaenium sp.	−	−	−	−	−	+	5.56
	Ankistrodesmus sp.	−	+	+	+	+	+	33.33
	Chlorella sp.	+	–	+	+	+	+	27.78
	Closterium sp.	+	+	+	+	+	+	88.89
	Cosmarium sp.	−	+	+	+	+	+	27.78
	Crucigenia sp.	−	+	−	−	−	+	11.11
	Crucigneiella sp.	−	+	+	+	+	+	27.78
	Dictyosphaerium sp.	−	–	+	+	+	+	27.78
	Eudorina sp.	−	+	−	−	−	−	5.56
	Haloshaera sp.	−	+	+	−	−	+	16.67
	Hyalotheca sp.	−	−	+	+	+	+	44.44
	Micrasterias sp.	−	−	−	−	−	+	5.56
	Monoraphidium sp.	+	+	+	+	+	+	33.33
	Oocystis sp.	−	−	+	+	+	+	61.11
	Pediastrum sp.1	+	+	+	+	+	+	94.44
	Pediastrum sp.2	+	+	+	+	+	+	66.67
	Pediastrum sp.3	–	+	+	+	+	+	27.78
	Scenedesmus sp.	+	+	+	+	+	+	33.33
	Selenastrum sp.	−	−	−	+	+	–	11.11
	Spirogyra sp.	+	+	+	+	+	+	66.67

	Staurastrum sp.1	−	+	+	−	+	+	22.22
	Staurastrum sp.2	−	−	+	−	−	−	5.56
	Staurodesmus sp.	−	−	−	−	−	+	5.56
	Tetraedron sp.1	+	+	+	+	−	+	33.33
	Tetraedron sp.2	−	+	+	+	−	+	33.33
	Volvox sp.	−	−	−	+	−	+	11.11
Charophyta	*Euglena* sp.	+	+	+	+	+	+	83.33
	Phacus sp.1	+	+	+	+	+	+	94.44
	Phacus sp.2	+	+	+	+	+	+	88.89
	Phacus sp.3	+	+	+	+	+	+	66.67
	Phacus sp.4	+	+	+	+	+	+	50.00
	Stombomonas sp.	−	+	+	+	+	+	38.89
	Trachelomonas sp.	−	+	+	+	+	+	27.78
Chrysophyta	*Centritractus* sp.	−	+	+	+	+	+	27.78
	Isthmochloron sp.	−	−	+	−	−	−	5.56
Bacillariophyta	*Bacillaria* sp.	−	+	+	+	+	+	27.78
	Brachysira sp.	−	−	+	+	+	+	27.78
	Cyclotella sp.	−	−	−	−	+	−	5.56
	Diploneis sp.	−	+	−	−	−	−	5.56
	Frustulia sp.	−	+	+	+	+	+	33.33
	Gomphonema sp.	−	+	+	+	+	+	38.89
	Gyrosigma sp.	−	+	+	+	+	+	55.56
	Navicula sp.	−	+	+	+	+	+	50.00
	Synedra sp.	−	−	+	+	+	−	16.67
Pyrrhophyta	*Ceratium* sp.	−	+	+	−	−	+	33.33
	Peridinium sp.	−	+	+	+	+	+	27.78
	**Total taxa**	**15**	**39**	**43**	**36**	**38**	**46**	

Table S3. Occurrence and relative frequency (*F*; %) of zooplankton in the fairy shrimp pond.

Categories	Scientific Name	Day 0	Day 3	Day 6	Day 9	Day 12	Day 15	*F*
Protozoa	*Arcella* sp.	–	–	+	+	+	+	66.67
	Difflugia sp.	–	+	+	+	+	–	33.33
Rotifera	*Anuraeopsis fissa*	+	–	–	–	–	–	11.11
	Anuraeopsis coelata	+	+	–	–	–	–	27.78
	Asplanchna brigthwellii	+	+	–	–	+	+	50.00
	Brachionus angularis	+	+	+	+	+	+	72.22
	Brachionus bidentatus	+	+	+	+	+	+	100.00
	Brachionus calyciflorus	+	+	+	–	–	+	44.44
	Brachionus caudatus	+	+	–	–	–	–	27.78
	Brachionus diversicornis	–	–	–	+	–	+	22.22
	Brachionus falcatus	+	+	+	+	+	+	77.78
	Brachionus forficula	+	+	–	–	+	+	50.00
	Brachionus murphyi	–	+	–	–	+	+	33.33
	Brachionus quandridentatus	+	+	+	+	+	+	66.67
	Brachionus rubens	–	–	+	+	+	+	44.44
	Euchlanis dilatata	+	–	+	+	+	+	61.11
	Euchlanis incisa	–	–	–	–	+	–	5.56
	Filinia longiseta	+	+	+	+	+	+	55.56
	Hexarthra mira	–	–	–	–	–	+	5.56
	Keratella cochlearis	–	–	–	+	–	+	16.67
	Keratella tropica	+	+	+	+	–	+	44.44
	Lecane bulla	+	+	+	+	+	+	55.56
	Lecane curvicornis	+	–	–	–	–	+	11.11
	Lecane closterocerca	–	+	+	+	+	–	22.22
	Lecane luna	+	–	+	+	+	+	44.44
	Lecane tenuiseta	–	–	+	–	+	+	22.22
	Lecane hamata	–	+	+	+	+	+	38.89
	Lecane segersi	+	+	+	–	–	–	27.78

	Lepadella patella	–	–	+	+	+	+	33.33
	Lepadella ovalis	+	–	+	+	–	–	22.22
	Lepadella rhomboides	–	–	–	–	+	–	5.56
	Polyarthra vulgaris	+	+	+	+	+	+	94.44
	Testudinella patina	+	+	+	+	+	+	72.22
	Trichocerca pusilla	+	+	–	–	+	+	55.56
	Trichocerca tenuior	–	–	+	–	–	–	5.56
Cladocera	*Ceriodaphnia cornuta*	–	–	+	+	+	+	50.00
	Diaphanosoma excisum	–	–	+	–	+	+	22.22
	Macrothrix spinosa	–	–	–	–	–	+	5.56
	Moina micrura	+	+	+	+	+	+	94.44
	Ovalona cambouei	–	–	+	+	+	+	50.00
	Pseudosida szalayi	–	–	+	–	–	–	5.56
	Scapholeberis kingi	+	+	+	+	+	+	55.56
	Simocephalus serrulata	–	–	–	+	–	–	5.56
Copepoda	*Mongolodiaptomus botulifer*	–	–	+	+	+	+	55.56
	Mongolodiaptomus calcarus	–	+	+	+	+	+	66.67
	Phyllodiaptomus praedictus	–	–	+	+	–	+	27.78
	Mesocyclops sp.	+	+	+	+	+	+	88.89
	Thermocyclops crasus	+	+	+	+	–	–	38.89
	Copepodite	+	+	+	+	+	+	100.00
	Nauplius	+	+	+	+	+	+	100.00
Ostracoda	Ostracods	–	+	–	+	+	+	55.56
	Total taxa	27	28	35	33	34	38	
